# Optimized Prediction of Reducing Sugars and Dry Matter of Potato Frying by FT-NIR Spectroscopy on Peeled Tubers

**DOI:** 10.3390/molecules24050967

**Published:** 2019-03-09

**Authors:** Cédric Camps, Zo-Norosoa Camps

**Affiliations:** Institute for Plant Production Sciences IPS, Agroscope, CH-1964 Conthey, Switzerland; zonorosoa@gmail.com

**Keywords:** Fourier-transform near-infrared spectroscopy, glucose, fructose, dry matter, partial least square regression

## Abstract

Dry matter content (DMC) and reducing sugars (glucose, fructose) contents of three potato varieties for frying (Innovator, Lady Claire, and Markies) were determined by applying Fourier-transform near-infrared spectrometry (FT-NIR), with paying particular attention to tubers preparation (unpeeled, peeled, and transversally cut tubers) before spectral acquisitions. Potatoes were subjected to normal storage temperature as it is processed in the industry (8 °C) and lower temperature inducing sugar accumulations (5 °C) for 195 and 48 days, respectively. Prediction of DMC has been successfully modeled for all varieties. A common model to the three varieties reached R^2^, root mean square error (RMSEP), and ratio performance to deviation (RPD) values of 0.84, 1.2, and 2.49. Prediction accuracy of reducing sugars was variety dependent. Reducing sugars were accurately predicted for Innovator (R^2^ = 0.84, RMSEP = 0.097, and RPD = 2.86) and Markies (R^2^ = 0.78, RMSEP = 0.033, and RPD = 2.15) and slightly less accurate for Lady Claire (R^2^ = 0.63, RMSEP = 0.036, and RPD = 1.64). The lack of accuracy obtained with the Lady Claire variety is mainly due to the tight variability in sugar content measured over the storage. Finally, the best preparation of the tuber from the point of view of the accuracy of the prediction models was to use the whole peeled potato. Such preparation allowed for the improvement in RPD values by 15% to 38% the RPD values depending on reducing sugars and 35% for DMC.

## 1. Introduction

The potato is the fifth most produced agricultural product with 388 million tons, behind sugar cane (1.84 trillion tons), corn (1.13 trillion tons), wheat (771 million tons), and rice (769 million tons) [1]. It can be consumed fresh, dehydrated, or fried as a snack.

Around the world, the potato crisp is a particularly popular snack. At the industrial level, the preparation of chips requires particular attention to the formation of acrylamide. This chemical compound is suspected to of being carcinogenic to humans, and its evolution in industrial products is closely monitored. During cooking, reducing sugars and asparagine interact can lead to browning on the outline and sometimes the whole of the chips, as a result of the reaction of Maillard [2]. The more the potato is rich in reducing sugars (glucose, fructose, and sucrose), the more the browning is visible. Whether for health or commercial reasons, the industry seeks to limit this browning. To limit the formation of acrylamide in food products, FoodDrinkEurope [3] has published tools in the form of "toolboxes" that are intended for the food industry. Among the first recommendations is the need to use potato varieties with low levels of reducing sugars. Some studies have already shown that there is a strong link between the reducing sugar content and the final level of acrylamide [4,5]. From both sanitary and aesthetic points of view, it is important for the industry to find an effective and accurate way to quantify the reducing sugars in their raw material, the fresh potato.

Indeed, the choice of a variety with low content of reducing sugars is a first big step, but there remains a consideration for an intra-varietal variability of reducing sugar content point of view. As a result, the method to be developed by the industry must be able to scan the largest number of individual tubers along the supply and processing chain.

During the last twenty years, near-infrared spectroscopy has been developed as a fast, precise, and mostly non-destructive method for the quality control of the agri-food sector. This method developed in the laboratory has the potential to be implemented in the industry for a chain analysis. Studies described the ability to quantify sugar content in fresh apples [6,7]. Subsequently, this method of measuring sugars has been extended to other fruits [8,9]. Studies have been conducted on potatoes to test the possibility of using near-infrared spectroscopy to measure the sugar or dry matter content of potatoes [10,11,12,13,14]. These studies are difficult to compare because some focused on whole tubers, whole and peeled, in cross-sections, or crushed in the form of puree. However, these studies have shown the potential of this method for application on the potato industry.

In general, the Swiss potato industry only accepts tubers with a reducing sugar content of less than 0.1% (*w*/*w*) for the manufacture of potato chips. The aim of the present study will be to develop predictive models for reducing sugars and dry matter content of fresh potato tubers based on Fourier-transform near-infrared spectroscopy (FT-NIRs). The novelty of this work is to use the advantages of Fourier-transform spectroscopy compared to spectroscopy from sequential instruments using monochromators or filters and multichannel instruments using diode arrays. The advantages of FT-NIR spectroscopy are significant, e.g., the usage of the interferometer saves time in acquiring spectra (Fellgett advantage) and allows higher throughput by passing through a larger amount of the NIR radiation, which can then be emitted or reflected by the sample (Jaquinot advantage) [15]. From the point of view of the potential future application in industry, it is important to note that FT-NIR spectroscopy has a lower sensitivity to stray light compared to monochromator and diode array devices. Such an advantage is significant since, in the industry, the environmental conditions can be more difficult to control compared to those at the lab [16]. Finally, these advantages are not detrimental to precision because the FT-NIR spectroscopy allows the highest precision of wavenumber (Connes’ advantage). FT-NIR spectrometers present the best wavelength precision, accuracy, high signal-to-noise, and scan speed [17]. In this way, the FT-NIR-based technique is fully capable of being transferred from a lab to the potato industry. To date, studies aiming at predicting dry matter and reducing sugars in potatoes have been carried out using monochromators or diode array-based instruments and often present a reduced range of NIR spectral absorbance (i.e., short-wave NIR wavelengths or higher than 1100 nm). The present study aims to highlight the potential of FT-NIR spectroscopy to predict dry matter and reducing the sugar content of potato tubers.

In order to optimize the method, three types of fresh tuber preparations were tested to decide the optimal preparation for maximum accuracy of the prediction models. Finally, the study worked on three varieties adapted to the potato industry, and the models were calibrated and validated with potato provided by industrials gathering tubers from different producers and different production sites.

## 2. Results and Discussion

### 2.1. Prediction of Dry Matter Content

Dry matter content (DMC) of individual potatoes was monitored during storage at 5 °C and 8 °C for the three varieties. Before storage, Lady Claire presented significantly (*p* < 0.0001) higher DMC values (26%) compared to Markies (23%) and Innovator (24%). This range of values is consistent with those found in several studies and in particular that of Elmore et al. [18] which compared the DMC of 20 UK-grown varieties intended for frying. The authors determined average values of DMC in a range of 17% to 28% depending on the variety, with Lady Claire being around 26%, and Innovator and Markies around 24%.

During storage at 5 °C, DMC remained steady while higher values were measured after 62 days at 8 °C for Lady Claire (*p* < 0.0001) and after 195 days at 8 °C for Markies (*p* < 0.0001) and Innovator (*p* < 0.0001). Finally, DMC values ranged from 18% to 40% depending on the variety and the storage modalities.

Then, DMC values were used to elaborate prediction models based on FT-NIR spectral data (Table 1). More than 400 and 100 spectra were used to calibrate and validate the models, respectively. Three models were attempted, with the first one based on spectra acquired on entire and unpeeled potatoes (PDTE), the second one with spectra of entire and peeled potatoes (PDTP), and the third one with spectra acquired on transversal cuts of potatoes (PDTC). The best result was obtained with spectra based on PDTP. This model presents the highest R^2^ value (0.84), and the lowest root mean square error (RMSE) value (1.23%).

**Figure 1:**
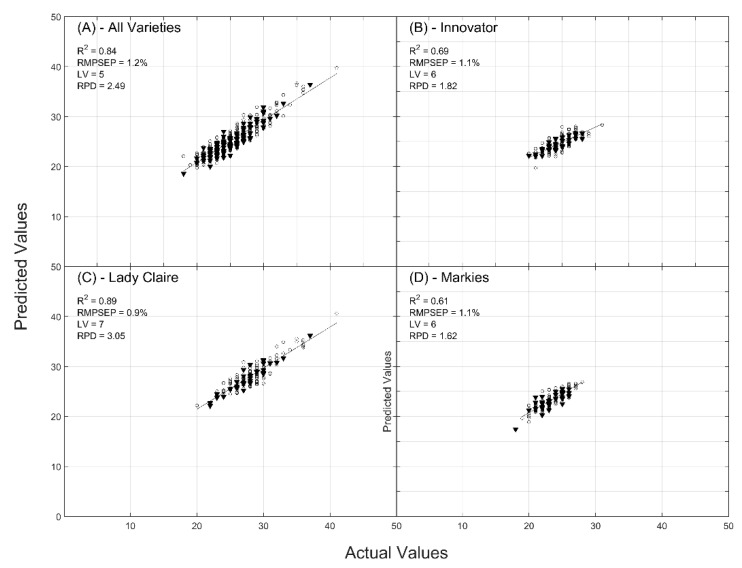
Actual vs. predicted values of dry matter content (DMC) (g of dry weight/100 g fresh weight). Calibration (○), validation (▲). (**A**) Entire and peeled tubers of the three tested varieties, (**B**) Entire and peeled tubers of Innovator, (**C**) Entire and peeled tubers of Lady Claire, (**D**) Entire and peeled tubers of Markies.

Furthermore, the model used only five latent variables while seven have been required for the PDTE- and PDTC-based models. In the present modeling, all potato varieties were successfully gathered for elaborating a “trans-varietal” model (Figure 1A). Modeling with separating the varieties allowed us to slightly improve the model performances of the Lady Claire variety in terms of ratio performance to deviation (RPD) value (3.05) (Figure 1C). Concerning the Innovator and Markies varieties, models were less accurate (Figure 1B,D).

The performance of the model gathering all varieties is comparable to that obtained by Helgerud et al. [19] who reached R^2^ and RMSE values of 0.8–0.9 and 0.9–1.7, respectively. However, the model developed with peeled potatoes only used five latent variables while the model developed by Helgerud et al. [19] used between five and nine latent variables. When the models were separately built per each variety, the accuracy increased until reaching R^2^ and RMSE values of 0.89 and 0.9%, respectively, for the Lady Claire variety. The performances were not improved for the two other varieties since the ranges of DMC values were very tight. Hartmann and Büning-Pfaue [20] were one of the first to study the prediction of DMC of potatoes using NIR spectral data acquired on peeled potato. They concluded that the accuracy of models was cultivar-dependent. In the present study, the feasibility of a model gathering the three cultivars shows that it is possible to predict DMC without developing a cultivar-dependent model. Subedi and Walsh [21] reported accurate predictions of DMC in potatoes using short-wave NIR spectral data with R^2^ values that ranged between 0.80 and 0.95 and RMSECV values that ranged from 0.5% to 1.52% depending on the cultivar. The authors used a batch of potatoes with a range of DMC values of 17% to 25%. The results obtained in the present study using FT-NIR spectroscopy allowed for obtaining similar accuracy for a range of DMC values of 18% to 40%.

DMC is crucial for the quality of chips or different forms of potato-based frites. Indeed, several studies showed the tight relationship existing between DMC and starch content [22,23]. Monitoring the DMC of fresh potatoes with FT-NIR spectroscopy could be an indirect indicator of starch content-related quality.

**Figure 2:**
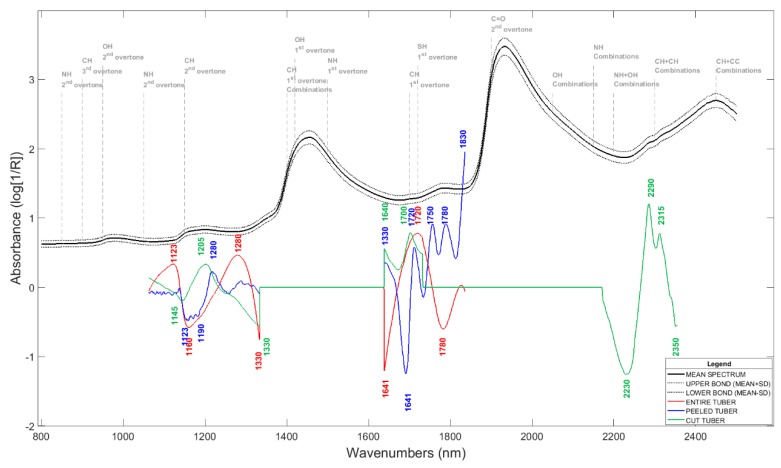
Mean spectra and beta-coefficients of the first PLS models latent variable to predict the DMC based on spectral data acquired on entire tubers (red line), peeled tubers (blue line), and cut tubers (green line).

Figure 2 shows the beta-coefficients of the first latent variable of the Partial least square models predicting the DMC of peeled, entire, and cut potatoes. Peeled potato model is based on two wavelength bands (1060–1330 nm and 1640–1830 nm). Such bands are mainly related to the second and first C–H overtones, respectively. Subedi and Walsh [21] identified the short-wave NIR region (750–950 nm) and particularly high importance of absorbance at 910 nm as significant to predict DMC in potatoes. This region is essentially related to absorption bands of the third overtone of CH and NH. In their study, the authors used a spectrometer whose wavelengths did not exceed 1100 nm, as they did not have access to the first overtones and combinations of CH and OH molecular bonds. In the present study, the two bands identified as significant in predicting DMC corresponded also to NH and CH molecular bonds, but these are the first and second overtones. The absorbance in the short-wave NIR range did not appear as significant. Finally, it can be considered that the main difference between the study of Subedi and Walsh [21] and the present study rely on the wavelength range availability due to the spectrometer. Hartmann and Büning-Pfaue [20] predicted DMC using a spectrometer with a wavelength range comprised between 1100 nm and 2500 nm. The accuracy of the models was correct, but no information about beta-coefficients was provided to determine the significance of wavelength absorbencies.

### 2.2. Prediction of Reducing Sugars

Reducing sugars were measured in individual potatoes during storage of the three varieties (Table 2). Sugar levels remained low (under 0.1%) over the 195 days of storage at 8 °C for all varieties. During storage at 5 °C, the levels of sugars were a function of the variety. Sugar contents increased slightly for Markies (0.14% fresh weight, FW) and Lady Claire (0.21% FW), and more strongly for the Innovator (0.70% FW) variety (Table 2).

Predictions of sugars contents were attempted for each potato variety. Prediction values of the Innovator, Lady Claire, and Markies varieties have been gathered in Table 3, Table 4 and Table 5. Predictions of glucose, fructose, and reducing sugars (glucose + fructose) were performed using FT-NIR data acquired on entire and unpeeled potatoes (PDTE), entire and peeled potatoes (PDTP), and transversally cut potatoes (PDTC).

**Figure 3:**
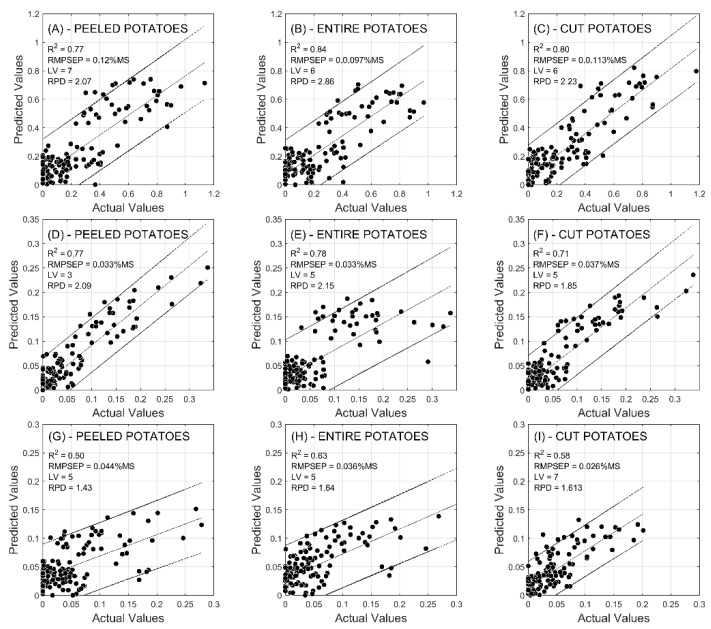
Actual vs. predicted values of Innovator’s (**A**–**C**), Markies’ (**D**–**F**) and Lady Claire’s (**G**–**I**) reducing sugars contents. Predictions were performed based on “Entire and peeled” (**A**,**D**,**G**) tubers, “Entire and unpeeled” tubers (**B**,**E**,**H**), and tubers “cut transversally” (**C**,**F**,**I**).

Potatoes preparation for FT-NIR spectra acquisition affected the overall performance of models (Figure 3).

PDTP (peeled potatoes) configuration allowed us to reach the most accurate models. Reducing sugar (fructose + glucose) were predicted with RPD values of 2.07 (PDTE), 2.86 (PDTP), and 2.23 (PDTC) for Innovator, 2.09 (PDTE), 2.15 (PDTP), and 2.1.85 (PDTC) for Markies, and 1.43 (PDTE), 1.64 (PDTP), and 1.61 (PDTC) for Lady Claire. In the same way, R^2^ and RMSE values were generally favored by the PDTP configuration (Table 3, Table 4 and Table 5).

In a study, Rady and Guyer [14] worked on the prediction of sugar by near-infrared spectroscopy using whole or sliced potatoes. They showed few differences between the two potato preparations from a model accuracy point of view. This result was similar in our study since the results obtained with PDTE and PDTC were quite similar and less accurate than those obtained with the PDTP. A reason why the PDTP was the most efficient method could be due to the fact that the reducing sugars are concentrated at the periphery of the tuber. On whole tuber, Chen et al. [24] predicted sugar contents with RMSE of 0.26 mg/g (0.026%) for fructose and 0.46 mg/g (0.046%) for glucose. For this, they used a range of NIR wavelength of 400–1100 nm. These results are comparable to ours, although the latter is strongly related to the variety and the total reducing sugar content of potatoes. Furthermore, Chen et al. [24] used particularly drastic conditions of storage, such as 25 °C storage temperature during several months. Such a storage parameter is not representative of the real storage rules for potatoes and induced very high sugar levels in tubers. Consequently, the tested storage conditions would be a simulation and consequence of at-home consumers’ storage rather than professional storage conditions. The very large range of sugar content allowed Chen et al. (2010) to develop a correct model in terms of accuracy. Rady and Guyer [14] obtained results showing the variability of model accuracy as a function of the used variety. They obtained R^2^values from 0.55 to 0.88 and RPD values from 1.49 to 2.73. However, it benefited from varieties with a greater range of sugar concentration than the varieties used in our study. Our lower sugar range gives our results a lot of room for improvement and the performance of our models could be increased by adding varieties with other ranges of sugar values.

In the present study, we considered that two of the three tested varieties (Innovator and Markies) were suitable for building a prediction model using near-infrared spectral data, but the last variety was inadequate (Lady Claire). The latter proved to be very insensitive to storage conditions and, as a result, the variations in sugar content were very small. However, this variety is not the right candidate to build our models, but it remains a good candidate for the French fries potato industry.

**Figure 4:**
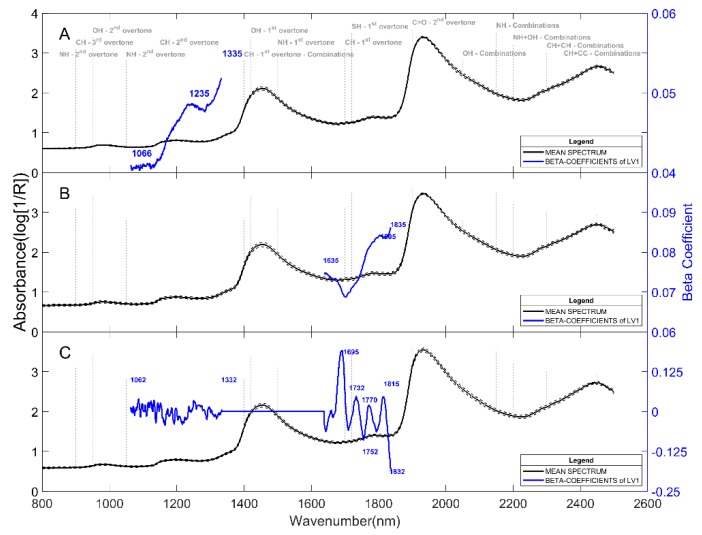
Mean spectra and beta-coefficients of the first PLS models latent variable to predict the reducing sugars of the three potato varieties: Innovator (**A**), Lady Claire (**B**), and Markies (**C**).

Figure 4 shows the beta-coefficients of the first latent variable of the PLS models predicting the reducing sugars of peeled tubers for the three varieties. Prediction of reducing sugars is based on wavelength bands between 1065 nm and 1335 nm, 1635 nm and 1835 nm, and both bands for Innovator, Lady Claire, and Markies varieties, respectively. Thus, models relied on second and first overtones of CH molecular bonds. Chen et al. [24], who used only a short-wave NIR range, identified relevant absorbencies in the vicinity of 710 nm (fourth overtone CH) and 888 nm (third overtone CH), but also bands around 950–960 nm (water band, second overtone OH) and 1020 nm (second overtone NH). The two first bands are consistent with the results obtained in the present study. The water bands were removed from the range used in our models (around 950 nm, 1450 nm, and 1900–1950 nm). The Relevance of wavelength absorbance at 910 nm to predict sugars in potatoes has been assigned by previous studies and is confirmed by the present FT-NIR approach [25]. The use of the full NIR range allowed for assigning other overtones of CH bonds to reduce sugar of potatoes. Finally, the FT-NIR approach suggests a possible transfer from the laboratory to the industry in the medium term. However, the models will still have to be enriched with other varieties of potatoes with different sugar content variations during storage to make the models more robust and confirm the relevant wavelengths already assigned.

## 3. Materials and Methods

### 3.1. Potatoes

Three potato varieties for frying were used in the present study: Lady Claire, Innovator, and Markies. In order to increase the variability of quality due to the varieties, the potatoes were stored into two different conditions. The first batch was stored in classical conditions at 8 °C and a second one at 5 °C to increase the sugar contents over time. A first batch of potatoes was analyzed some days after harvest. Then, potato batches stored at 5 °C were analyzed after 7, 21, and 49 days while potatoes stored at 8 °C were analyzed after 7, 62, and 195 days. A given batch was constituted of 25 potatoes per variety. A total of 525 tubers were analyzed.

### 3.2. Dry Matter Content

A sample of each fresh potato (a cube of about 2 cm on each side) was weighed to obtain the fresh weight (FW). Then, the sample was placed in a dryer at 70 °C for 6 days and weighed again to obtain the dry weight (DW). The relative content of dry matter (DMC) was calculated according to Equation (1).

(1) $$\mathrm{DMC}\left(\%\right)=\left[1-\left(\frac{\mathrm{FW}\left(g\right)-\mathrm{DW}\left(g\right)}{\mathrm{FW}\left(g\right)}\right)\right]\times 100$$

### 3.3. Reducing Sugars Analyses

Sugar analyses were performed using enzymatic tests (Enzytec™ Fluid D-Glucose, r-Biopharm, Darmstadt, Germany). The limit of detection (LoD) and quantification (LoQ) calculated according to the method DIN 32645:2008-11 were 4.0 mg/L and 10 mg/L, respectively (Enzytec™ Fluid D-Glucose, r-Biopharm, Germany). A repeatability calculation was performed on 19 potato samples and duplicated. Relative standard errors of 1.6% and 1.7% were calculated for the glucose and fructose, respectively.

#### 3.3.1. Extraction

Approximately 1 g of the potato powder was weighed into a 50 mL centrifuge tube. A total of 10 mL of ethanol at 40% was added. The mixture was then extracted for 1 min using an Ultra-Turrax (Polytron PT3100/Polytron PT 10 20 3500, Kinematica AG, Luzern, Switzerland) on the highest rpm setting. After that, the tubes were centrifuged for 5 min at 4000 rpm. The extract was filled into a 50 mL measuring flask. This extraction was repeated twice, and extracts were pooled together. Tubes were then filled to the calibration mark with ethanol at 40%.

#### 3.3.2. Quantification of Free Sugars

A total of 10 mL of the ethanol extract was pipetted into a 25 mL pointed flask. The solvent was then evaporated using a Rotavap at 60 °C. The residue was solved in 2 mL of distilled water. The results were expressed in milligrams of sugar contained in 100 g of potato fresh weight. An aliquot of the sample was filtered through a 0.45 µm syringe filter (nylon). Glucose/fructose contents of this filtered sample were quantified by photometric methods using the Konelab Arena 20XT (Thermo Fisher Scientific OY, Vantaa, Finland).

### 3.4. FT-NIR Spectroscopy

FT-NIR measurements (MPA, Bruker, Fällanden, Switzerland) were carried out according to 3 configurations, all using an optical fiber. Spectral acquisitions were performed on (1) entire and unpeeled potatoes (PDTE), (2) entire and peeled potatoes (PDTP), and (3) potatoes cut transversally (PDTC). Spectra were acquired in diffuse reflexion using an optic fiber. A total of 3 spectra were recorded per potato. A given spectra was the average of 16 scans in a wavenumber range comprised between 12,500 cm^−1^ and 4000 cm^−1^. A total of 4725 spectra was collected in the present study (4725 = (25 potatoes) × (3 varieties) × (7 batches of storage) × (3 spectra per potato) × (3 configurations of spectra measurements)). All spectra were collected with the OPUS software (Bruker, Germany).

### 3.5. Chemometric

#### Partial Least Square Regression

Averaged spectra of each tuber were gathered in a matrix X(n,p) where ‘n’ is the number of spectra and ‘p’ is the number of wavenumber steps. The reference values (sugars) were gathered in a column vector y(n,1). Potato batches were separated in a calibration set (3n/4) and a test set (n/4). The accuracy and goodness of models were evaluated according to several indicators: the coefficient of determination (R^2^), root mean square errors (RMSE), and the ratio performance to deviation (RPD) [15]. All data analyses were performed with OPUS software and Matlab R2016a (The MathWorks, Inc., Natick, MA, USA).

## 4. Conclusions

The potato industry needs to precisely monitor the quality of the potatoes along the supply chain to ensure the optimal quality of the final product both in terms of aesthetics and sanitation. The Fourier-transform near-infrared spectroscopy is a possibility since this technology is already implemented in various food industries and presents significant advantages compared to monochromator and diode array-based NIR instruments. The study presented in this paper aimed to evaluate the possibility of determining the dry matter and reducing sugars contents in fresh potatoes which are the primary matters of the industry. The results obtained are promising in terms of accuracy. In addition, sample preparation is important when working with NIR spectroscopy. The present study tested three different tuber preparations in order to optimize the configuration of spectral acquisitions. The peeled but not necessarily crushed potato was determined to be the most interesting. This preparation has made it possible to obtain more precise models for sugar and dry matter contents. In particular, it has improved the RPD values from 15% to 38% for reducing sugars and 35% for DMC. Finally, since the robustness of the models is closely linked to the variability introduced, additional potato varieties adapted to the potato frying industry should be added to the present models. In addition, different storage conditions may be important to make the models more robust.

## Figures and Tables

**Table 1:** Partial least square values of dry matter content prediction. PDTE: entire and unpeeled potatoes, PDTP: entire and peeled potatoes; PDTC: potatoes cut transversally.

PLS Parameters		PDTE	PDTP	PDTC
Spectra (n)	CAL/VAL	420/105	417/104	417/103
Averaged spectra (n)	CAL/VAL	140 (140) ^1^/35	139 (140) ^1^/35	140 (140) ^1^/35
Wavenumber range (cm^−1^)		9403.8–7498.4 6102.1–5446.3	9403.8–7498.4 6102.1–5446.3	9403.8–7498.4 6102.1–5774.2 4601.6–4246.8
LV	CAL/VAL	7/7	7/7	7/7
R^2^	CAL/VAL	0.76/0.70	0.84/0.83	0.83/0.78
RMSE (%)	CAL/VAL	1.59/1.66	1.27/1.23	1.34/1.40
RPD	CAL/VAL	2.05/1.85	2.55/2.49	2.45/2.17
**Data preprocessing**	**CAL/VAL**	**LOS**	**D1 + SNV**	**SNV**
DMC values (min–max) (**g_DW_/100 g_FW_**)	CAL/VAL	18–41/18–37	18–41/18–37	18–40/18–37
DMC Standard error (%)	CAL/VAL	3.22/3.07	3.22/3.07	3.22/3.07

^1^ number of averaged spectra (tubers) before elimination of outlayers. CAL: calibration; VAL: validation; n: number of spectra or potato samples; LV: the number of latent variables; R^2^: determination coefficient; RMSE: root mean square error; RPD: ratio performance to deviation; DMC (%): range of dry matter content values used in the models; DW: dry weight; FW: fresh weight. LOS: Linear offset subtraction; SNV: Standard Normal Variate; D1: first derivative.

**Table 2:** Sugar values of individual potatoes. Sugar values were statistically analyzed by the non-parametric test of Kruskal–Wallis (*p* = 0.05); mean values comparisons have been processed by the Dun test. RS: Reducing sugars, FW: fresh weight.

Duration (Days)	Temp. (°C)	Glucose (% FW)			Fructose (% FW)			RS (Glucose + Fructose) (% FW)		
		IN	LC	MA	IN	LC	MA	IN	LC	MA
0	-	0.07 a,b	0.03 c	0.02 b	0.05 b,c	0.01 b,c	0.01 b	0.11 a,b	0.04 b	0.04 b
6	5	0.11 b	0.03 c	0.02 b	0.09 c	0.03 c,d	0.02 b	0.20 b	0.06 b	0.04 b
24	5	0.30 c	0.05 c,d	0.08 c	0.27 d	0.05 d,e	0.10 c	0.57 c	0.10 b,c	0.18 c
48	5	0.37 c	0.07 d	0.10 c	0.33 d	0.07 e	0.11 c	0.70 c	0.14 c	0.21 c
6	8	0.08 a	0.02 b,c	0.02 b	0.06 b,c	0.01 c,d	0.01 b	0.14 a,b	0.03 b	0.03 b
62	8	0.03 a	0.01 a,b	0.01 a,b	0.02 a,b	0.00 a,b	0.01 a,b	0.06 a	0.01 a	0.01 a,b
195	8	0.04 a	0.00 a	0.00 a	0.01 a	0.00 a	0.00 a	0.05 a	0.01 a	0.00 a
*p*-value		<0.0001	<0.0001	<0.0001	<0.0001	<0.0001	<0.0001	<0.0001	<0.0001	<0.0001

Means followed by different letters within one column differ significantly at *p* = 0.05.

**Table 3:** PLS values of sugar content prediction of the potato variety Innovator.

PLS Score		Glucose			Fructose			Fructose + Glucose		
		PDTE	PDTP	PDTC	PDTE	PDTP	PDTC	PDTE	PDTP	PDTC
Spectra	CAL	420	405	405	402	402	405	405	405	408
	VAL	105	102	99	99	105	99	102	105	99
Averaged spectra	CAL	140 (140) ^1^	135 (140)	135 (140)	134 (140)	134 (140)	135 (140)	135 (140)	135 (140)	136 (140)
	VAL	35 (35)	34 (35)	33 (35)	33 (35)	35 (35)	33 (35)	34 (35)	35 (35)	33 (35)
WL (cm^−1^)		9403.8–7498.4; 6102.1–5446.3	9403.8–7498.4; 5774.2–5446.3	9403.8–7498.4	8451.1–7498.4; 5774.2–5446.3	9403.8–7498.4	9403.8–7498.4	9403.8–7498.4; 6102.1–5446.3	9403.8–7498.4	9403.8–7498.4
LV		7	6	7	8	6	6	7	6	6
R^2^	CAL	0.56	0.71	0.82	0.72	0.71	0.77	0.68	0.69	0.76
	VAL	0.70	0.82	0.79	0.84	0.82	0.85	0.77	0.84	0.80
RMSE (%)	CAL	0.108	0.076	0.061	0.065	0.071	0.061	0.149	0.151	0.129
	VAL	0.084	0.059	0.066	0.050	0.050	0.049	0.120	0.097	0.113
RPD	CAL	1.51	1.87	2.35	1.9	1.87	2.08	1.75	1.8	2.05
	VAL	1.89	2.34	2.25	2.5	2.43	2.59	2.07	2.86	2.23
**Data Preprocessing**		**SNV**	**SNV**	**LOS**	**MMN**	**None**	**LOS**	**MMN**	**None**	**LOS**
Sugar values (% FW)	CAL	0–0.709	0–0.596	0–0.613	0–0.417	0–0.539	0–0.564	0–0.969	0–1.135	0–1.178
	VAL	0–0.494	0–0.476	0–0.493	0–0.39	0–0.39	0–0.39	0–0.779	0–0.779	0–0.779

^1^ number of averaged spectra (tubers) before elimination of outlayers. CAL: calibration; VAL: validation; spectra: number of spectra or potato samples; LV: the number of latent variables; R^2^: determination coefficient; RMSE: root mean square error; RPD: ratio performance to deviation; SNV: Standard Normal Variate; LOS: Linear offset subtraction; MMN: Min–Max Normalization.

**Table 4:** PLS values of sugar content prediction of the potato variety Markies.

PLS Score		Glucose			Fructose			Fructose + Glucose		
		PDTE	PDTP	PDTC	PDTE	PDTP	PDTC	PDTE	PDTP	PDTC
Spectra	CAL	402	396	405	405	405	405	411	405	405
	VAL	102	102	102	102	99	99	102	99	102
Averaged spectra	CAL	134 (140) ^1^	132 (140)	135 (140)	135 (140)	135 (140)	135 (140)	137 (140)	135 (140)	135 (140)
	VAL	34 (35)	34 (35)	34 (35)	34 (35)	33 (35)	33 (35)	34 (35)	33 (35)	34 (35)
WL (cm^−1^)		8451.1–7498.4	9403.8–8451.1; 6102.1–5774.2	9403.8–8451.1; 6102.1–5446.3	8451.1–7498.4	9403.8–8451.1; 6102.1–5774.2	9403.8–7498.4; 5774.2–5446.3	8451.1–7498.4	9403.8–7498.4; 6102.1–5446.3	9403.8–7498.4; 5774.2–5446.3
LV		5	9	4	3	9	9	3	5	5
R^2^	CAL	0.71	0.83	0.72	0.61	0.83	0.88	0.55	0.81	0.78
	VAL	0.80	0.75	0.70	0.75	0.81	0.77	0.77	0.78	0.71
RMSE (%)	CAL	0.019	0.013	0.019	0.022	0.016	0.013	0.051	0.031	0.034
	VAL	0.015	0.017	0.019	0.018	0.016	0.017	0.033	0.033	0.037
RPD	CAL	1.86	2.4	1.89	1.6	2.39	2.94	1.5	2.31	2.14
	VAL	2.29	2.14	1.83	2.01	2.4	2.11	2.09	2.15	1.85
**Data Preprocessing**		**SLS**	**None**	**MSC**	**1st der.**	**None**	**None**	**1st der.**	**D1 + MSC**	**MSC**
Sugar values (% FW)	CAL	0–0.165	0–0.128	0–0.165	0–0.173	0–0.173	0–0.173	0–0.338	0–0.338	0–0.338
	VAL	0–0.115	0–0.115	0–0.115	0–0.119	0–0.119	0–0.119	0–0.235	0–0.235	0–0.235

^1^ number of averaged spectra (tubers) before elimination of outlayers. CAL: calibration, VAL: validation; spectra: number of spectra or potato samples; LV: the number of latent variables; R^2^: determination coefficient; RMSE: root mean square error; RPD: ratio performance to deviation; MSC: Multiplicative scatter correction; 1st der.: first derivative; SLS: Straight line subtraction.

**Table 5:** PLS values of sugar content prediction of the potato variety Lady Claire.

PLS Score		Glucose			Fructose			Fructose + Glucose		
		PDTE	PDTP	PDTC	PDTE	PDTP	PDTC	PDTE	PDTP	PDTC
Spectra	CAL	396	393	408	408	411	411	414	411	393
	VAL	96	105	102	99	105	102	102	105	96
Averaged spectra	CAL	132 (140) ^1^	131 (140)	136 (140)	136 (140)	137 (140)	137 (140)	138 (140)	137 (140)	131 (140)
	VAL	32 (35)	35 (35)	34 (35)	33 (35)	35 (35)	34 (35)	34 (35)	35 (35)	32 (35)
WL (cm^−1^)		7502.2–6098.2	9403.8–7498.4	9403.8–7498.4; 6102.1–5446.3	9403.8–6098.2	9403.8–7498.4; 4601.6–4246.8	9403.8–7498.4; 6102.1–5446.3	9403.8–7498.4	6102.1–5446.3	9403.8–7498.4; 6102.1–5446.3
LV		6	5	6	9	14	6	5	5	7
R^2^	CAL	0.55	0.57	0.61	0.57	0.64	0.59	0.45	0.38	0.64
	VAL	0.57	0.52	0.61	0.67	0.70	0.63	0.50	0.63	0.58
RMSE (%)	CAL	0.015	0.018	0.018	0.020	0.019	0.018	0.045	0.047	0.028
	VAL	0.016	0.014	0.018	0.016	0.017	0.018	0.044	0.036	0.026
RPD	CAL	1.49	1.52	1.6	1.74	1.68	1.56	1.35	1.27	1.66
	VAL	1.56	1.47	1.62	1.52	1.83	1.66	1.43	1.64	1.61
**Data Preprocessing**		**D1 + SNV**	**LOS**	**SLS**	**MMN**	**D1**	**SNV**	**SNV**	**None**	**LOS**
Sugar values (% FW)	CAL	0–0.103	0–0.131	0–0.131	0–0.138	0–0.147	0–0.138	0–0.300	0–0.279	0–0.202
	VAL	0–0.102	0–0.077	0–0.117	0–0.122	0–0.122	0–0.122	0–0.239	0–0.239	0–0.144

^1^ number of averaged spectra or potatoes before elimination of outlayers. CAL: calibration, VAL: validation; spectra: number of spectra or potato samples; LV: the number of latent variables; R^2^: determination coefficient; RMSE: root mean square error; RPD: ratio performance to deviation; SNV: Vector normalization; LOS: Linear offset subtraction; MMN: Min–Max Normalization; D1: first derivative; SLS: Straight line subtraction.

## References

[1] FAOSTAT Food and Agriculture Organization of the United Nations. http://www.fao.org/faostat/fr/#data/QC.

[2] Maillard L.C. (1912). Action des acides aminés sur les sucres: formation des mélanoïdines par voie méthodique. C. R. Hebd. Séances Acad. Sci..

[3] FoodDrinkEurope A “Toolbox” for the Reduction of Acrylamide in Fried Potato Crisps. https://www.fooddrinkeurope.eu/uploads/publications_documents/crisps-EN-final.pdf.

[4] Matthäus B., Haase N.U., Vosmann K. (2004). Factors affecting the concentration of acrylamide during deep-fat frying of potatoes. Eur. J. Lipid Sci. Technol..

[5] Williams J.S.E. (2005). Influence of variety and processing conditions on acrylamide levels in fried potato crisps. Food Chem..

[6] McGlone V.A., Jordan R.B., Seelye R., Clark C.J. (2003). Dry-matter-a better predictor of the post-storage soluble solids in apples?. Postharv. Biol. Technol..

[7] Peirs A.S.N., Touchant K., Nicolaï B.M. (2002). Comparison of Fourier transform and dispersive near-infrared reflectance spectroscopy for apple quality measurements. Biosys. Engineer..

[8] Garcia-Jares C.M., Medina B. (1997). Application of multivariate calibration to the simultaneous routine determination of ethanol, glycerol, fructose, glucose and total residual sugars in botrytized-grape sweet wines by means of near-infrared reflectance spectroscopy. Fresen. J. Anal. Chem..

[9] Camps C., Christen D. (2009). Non-destructive assessment of apricot fruit quality by portable visible-near infrared spectroscopy. Lwt-Food Sci. Technol..

[10] Mehrübeoǧlu M., Coté G.L. (1997). Determination of total reducing sugars inpotato samples using near-infrared spectroscopy. Cereal Food. World.

[11] Van Dijk C., Fischer M., Holm J., Beekhuizen J.G., Stolle-Smits T., Boeriu C. (2002). Texture of cooked potatoes (*Solanum tuberosum*). 1. Relationships between dry matter content, sensory-perceived texture, and near-infrared spectroscopy. J. Agric. Food Chem..

[12] Chen J.Y., Miao Y., Zhang H., Matsunaga R. (2004). Non-destructive determination of carbohydrate content in potatoes using near infrared spectroscopy. J. Near Infrared Spec..

[13] Haase N.U. (2011). Prediction of Potato Processing Quality by near Infrared Reflectance Spectroscopy of Ground Raw Tubers. J. Near Infrared Spec..

[14] Rady A.M., Guyer D.E. (2015). Evaluation of sugar content in potatoes using NIR reflectance and wavelength selection techniques. Postharvest Biol. Tec..

[15] Jacquinot P. (1984). How the search for a throughput advantage led to Fourier transform spectroscopy. Infrared Phys..

[16] White R. (1989). Chromatography/Fourier Transform Infrared Spectroscopy and Its Applications.

[17] Fernández Pierna J.A., Manley M., Dardenne P., Downey G., Baeten V., Sun D.-W. (2018). Spectroscopic Technique: Fourier Transform (FT) Near-Infrared Spectroscopy (NIR) and Microscopy (NIRM). Modern Techniques for Food Authentication.

[18] Elmore J.S., Briddon A., Dodson A.T., Muttucumaru N., Halford N.G., Mottram D.S. (2015). Acrylamide in potato crisps prepared from 20 UK-grown varieties: Effects of variety and tuber storage time. Food Chem..

[19] Helgerud T., Wold J.P., Pedersen M.B., Liland K.H., Ballance S., Knutsen S.H., Rukke E.O., Afseth N.K. (2015). Towards on-line prediction of dry matter content in whole unpeeled potatoes using near-infrared spectroscopy. Talanta.

[20] Hartmann R., Büning-Pfaue H. (1998). NIR determination of potato constituents. Potato Res..

[21] Subedi P.P., Walsh K.B. (2009). Assessment of Potato Dry Matter Concentration Using Short-Wave Near-Infrared Spectroscopy. Potato Res..

[22] Stark J.C., Love S.L. (2003). Tuber quality. Potato Prod. Sys..

[23] Storey R.M.J., Vreugdenhil D. (2007). The harvested crop. Potato Biology and Biotechnology Advances and Perspectives.

[24] Chen J.Y., Zhang H., Miao Y., Asakura M. (2010). Nondestructive determination of sugar content in potato tubers using visible and near infrared spectroscopy. Jpn. J. Food Eng..

[25] Yaptenco K.F., Suzuki T., Kawakami S., Sato H., Takano K., Kozima T.T. (2000). Nondestructive determination of sugar content in ’Danshaku’ potato (*Solanum tuberosum* L.) by near infrared spectroscopy. J. Agric. Sci..

